# Cross-Currents in the General Nursing Council

**Published:** 1920-12-18

**Authors:** 


### Cross-currents in the General Nursing Council.

The problem of nurses' hours is still unsettled, and if the Minister of Health holds strong views on the subject, he has received practically *carte blanche* to carry them into effect. For the General Nursing Council has quite evidently not yet found itself. It remains so far what it was at the beginning, an agglomeration of nominees appointed to represent various interests. By the resolution of the October meeting the Council voted against nurses being included in the Minister of Labour's Bill to regulate hours of employment. In November, on the motion of Dr. Bedford Pierce, they voted in favour of requesting the Minister of Health to introduce a Bill himself to regulate nurses' hours. It now appears that there is a distinct cleavage in the Council, some members advocating, others opposing the inclusion of private nurses in any Hours of Employment Bill for the profession. But this is not all.

**Matrons' and Sisters' Department**—(*continued*).

In spite of the resolution already passed desiring the Minister of Health to regulate nurses' hours, there are members of the Council who desire the rescission of the November resolution.

Mr. Christian's position, as representative of mental nursing interests, is quite sound and comprehensible. Mental nurses have consolidated their interests with those of asylum workers generally, and are strongly impelled to claim the protection of the Ministry of Labour. They have never vacillated about this. They are emerging from very hard conditions of service, and are mistrustful of what professional status can do for them apart from combination with other officials. It is for the Minister of Health to prove to them that under his ægis their interests will be fully safeguarded.

The vacillation about private nurses still continues. It is hardly credible that the Minister of Health will consent to leave outside and unprotected this large and influential class of women when protecting other nurses from the rapacity of employers. No nurses in institutions, public health or district work are so hardly used as private nurses in respect of their hours on duty.

The General Nursing Council at their meeting last week reaffirmed their resolution (1) that nurses should be excluded from the Hours of Employment Bill now before the House; (2) That the Minister of Health should be asked to bring in a Bill dealing with nurses' hours. Until the Minister replies to this request the Council refrains from entering more particularly into the question of institutional, district and private nursing.

